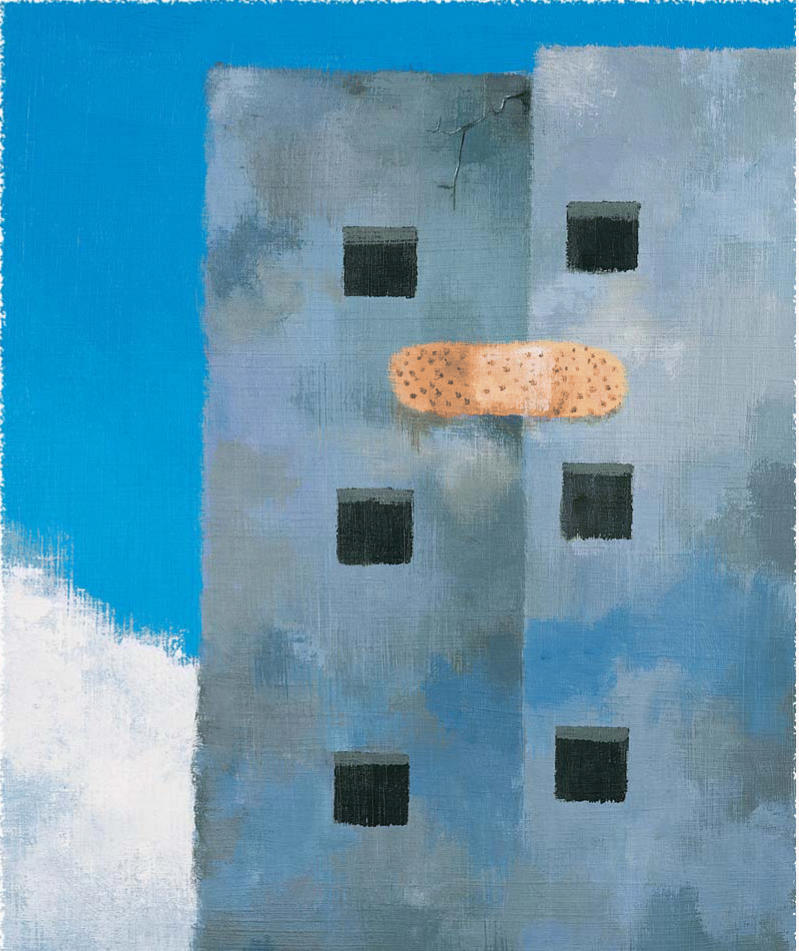# On Closer Inspection: Learning to Look at the Whole Home Environment

**DOI:** 10.1289/ehp.113-a320

**Published:** 2005-05

**Authors:** Angela Spivey

It’s supposed to be a harbor, a haven—it’s home. But some home environments can cause serious health problems. Moisture and molds can inflame asthma and allergies. Broken steps can cause a fall. A leaky oil furnace can produce deadly carbon monoxide. Cockroaches and mice can produce airborne allergens that trigger asthma episodes. A variety of health professionals and inspectors may enter a home for one reason or other, but few are equipped to spot all the possible ways a house can hurt its occupants. Now a new government partnership aims to change that.

To holistically address all the aspects of housing that affect health, the Centers for Disease Control and Prevention (CDC) and the U.S. Department of Housing and Urban Development have partnered to sponsor the National Healthy Homes Training Center and Network. Operated by the nonprofit National Center for Healthy Housing (NCHH), the network aims ultimately to change how city and state governments conduct public health activities related to housing.

The NCHH provides training tools and curricula, while the actual training is carried out by a nationwide network of universities including Eastern Kentucky University, The Johns Hopkins University, the University of Cincinnati, and the University of Washington. Rebecca Morley, executive director of the NCHH, hopes to expand the network soon to include other sites, such as Boston University and Boston Medical Center. “We made a conscious decision to use the already-trusted resources in the community,” Morley says. “By building on the existing infrastructure, we think we stand a lot better chance of bringing about change.”

Morley adds, “The role of the National Center for Healthy Housing is to anchor this network by providing training curricula and tools and serving as a repository for all the information about healthy housing that the partners might need to carry out the training and to promote healthy housing more broadly in their communities.” Part of that repository will be an electronic database of research information, assessment tools, and treatment protocols that will be available online in summer 2005.

So far the network has trained 25 workers through a 2004 pilot training session. This year, the goal is to expand training to at least four other training sites. By September 2006, the training will have reached nearly 600 public health nurses, home inspectors, weatherization inspectors, environmental health specialists, and others, Morley says.

## A New Use for Resources

The idea that housing and health are intertwined is not new. As far back as the 1800s, the close connection between the two earned government attention in the United States because of high rates of infectious disease in overcrowded city slums, which often had inadequate sewage treatment and a lack of running water.

Jerry Hershovitz, associate director for program development at the CDC’s National Center for Environmental Health and the Agency for Toxic Substances and Disease Registry, says that as recently as the 1960s, several large cities actually trained their public health staff across multiple disciplines. But as budgets eroded, health departments tended to become much more focused on narrow issues, largely because funding has tended to target single-issue programs such as pest control or lead hazards. Training, as well, has tended to be narrowly focused, says Morley. As a result, the public health and housing fields have grown increasingly specialized.

But as public health problems that were once emergencies have become more manageable and the infrastructures developed to address them have matured, policy makers have begun to take a broader look at housing health, says Ellen Tohn, president of the Wayland, Massachusetts, environmental consulting firm ERT Associates. The NCHH itself, for example, was formerly the National Center for Lead-Safe Housing; it changed its name in 1999 to reflect its increasingly broader scope. Grassroots groups have also played a role in the growing focus on a holistic approach to housing and health. For example, New England’s nonprofit Asthma Regional Council has worked with the building industry to create detailed guidelines for construction practices that can reduce conditions that trigger asthma.

In the last 10 years, the CDC has begun to revisit a holistic approach to healthy housing. “We believe that it’s more cost-effective and more cost-efficient in the long run to take a comprehensive approach towards the problem,” Hershovitz says. “The impact of housing on health and safety has emerged as a major public health concern. The whole basis for the initiative was to encourage public health programs to address multiple housing deficiencies and hazards that affect the health and safety of residents.”

## One-Touch System

Oftentimes, health departments receive funding for a single program to address a single issue. Individual workers will enter a house to investigate an isolated problem such as a child’s rat bite, peeling lead-based paint, or mold. But the training network is promoting a “one-touch” system in which one visit from a health or housing worker may trigger efforts to address multiple problems. With this one-touch system, anytime a health or housing worker enters a house, he or she will not only treat the problem that spurred the visit, but also will look for other housing problems that can threaten health, then refer the resident to someone who can fix the problem.

Such a holistic approach to healthy housing can make a real difference in people’s lives. For instance, in a pilot program in Philadelphia, environmental health workers working in rodent control carry carbon monoxide detectors and use them whenever they visit a house. “In the pilot area, they may have saved the lives of several people by doing that,” Hershovitz says.

Similarly, through a Boston pilot program called Breathe Easy at Home, any health care provider who treats someone with asthma and finds out there may be asthma triggers in the housing environment can make a special referral to the housing department of the Boston Public Health Commission. A specially trained inspector then inspects the patient’s home for conditions that exacerbate asthma and can cite violations and get such problems fixed. In one case, this program resulted in housing repairs that tenants had been requesting for a year, with significant reduction of a child’s asthma symptoms, Tohn says.

To bring more health departments nationwide on board with this one-touch model, the network organizers are counting on the frontline workers who actually visit people’s homes. “We’re trying to encourage each worker to think more broadly about what his or her role is,” says Morley. “When public health nurses walk into homes, immediately they focus on the people. We want to train all practitioners to look around the housing environment systematically to see what might be causing health problems.”

## A Broad Use of Basics

The training sessions offered by the new network include basic background information on environmental public health, building science, and specific housing-related hazards. This approach will push workers to reach outside their disciplines. Most public health nurses, for example, have not been exposed to the basics of housing construction. The training will include basic information about such features as site grading, drainage, and ventilation, all of which can affect health by causing excess moisture and poor air quality in the home. While a public health nurse wouldn’t be expected to solve a ventilation problem, this training would make the nurse better able to see the signs of poor ventilation and know where to refer the client.

Likewise, although most housing inspectors are versed in their local building codes, they may not always think of all the ways they can use the codes to get the most important health hazards repaired. For example, the building codes of most U.S. cities do not explicitly mention mold as a violation, says Tohn. “But every large city has a clause that will allow you to cite mold—it could be cited as a public nuisance, or as a failure of a building system, or as chronic dampness and moisture.” The training teaches workers which housing problems are most dangerous to occupants’ health and how to apply local codes to correct those problems. Participants also learn basic assessment and treatment skills such as testing for carbon monoxide and identifying mold and its causes.

The heart of the training is teaching workers to think more broadly. Rather than thinking in such narrow categories as lead abatement or asthma treatment, workers are taught to visualize their jobs as ensuring that houses are clean, dry, pest-free, safe, comfortable, well-ventilated, free of contaminants, and well-maintained. Tohn, who helped develop the curriculum, calls this checklist a mantra that ideally all front-line workers would keep in mind when they visit a home for any reason.

## Know Your Audience—And Your Colleagues

The training sessions also emphasize the importance of careful listening and observation. Casual conversation with residents can yield clues about where health threats might lurk in a home. “The training reminds workers to listen for those clues,” Tohn says. “Residents might say, ‘This is my brother-in-law. He’s living with us, but he hasn’t been able to work much.’ ‘Why?’ ‘He’s having trouble with asthma; he started having it after he moved in with us.’” Further conversation may reveal that the brother is sleeping in a room with poor ventilation or where there are cockroach or rodent droppings, any of which could trigger his asthma.

Workers should also be aware of how residents’ behaviors might be affecting the health environment of their homes. Terry Brennan, president of the New York–based building research and training firm Camroden Associates, points to the example of families who have immigrated to the United States from a tropical climate, who will try to recreate the high levels of indoor humidity they are accustomed to. “I find families who put pots of water on the stove and crank them up, and that gets the humidity level up to sixty or seventy percent inside the house,” he says. “But you can’t do that in, say, Minnesota; if it’s cold outside, you end up with condensation all over the walls and closets, which results in mold growth.”

It is not possible for the new network to train every frontline worker in the country, but Morley hopes this widespread training network will act as a catalyst. “The idea is for this training to become sustainable—we want it to become incorporated into the academic training process as well as into other existing training programs,” she says. “And we hope that the folks we train will go back to their departments and promote this systematic approach to housing.”

Another goal of the training sessions is to create connections among frontline workers in different fields. Even though a public health worker and a housing worker, for instance, are pursuing similar goals—including improving the health of housing residents—they often go about it very differently and rarely have a chance to meet. The sessions will therefore use small-group exercises to promote discussion among specialists from different fields.

Brennan, who helped conduct the pilot training, says that one of the most innovative aspects of the training—the way it brings housing workers and public health workers together in the same room—is also challenging because the curriculum must target people who have different sets of skills. But the two groups can learn from each other. “The public health folks know about our biggest health problems, what’s troubling us most now,” Brennan says. “The building folks know how buildings are made and how they fail.”

## Taking It into the Field

The participants in the 2004 pilot session were enthusiastic about a more holistic approach to healthy housing, but they pointed out that to achieve real change, they would need the support of supervisors and higher-level administrators. In response, the training center is developing a video and other materials targeted to decision makers to pave the way for their staff to follow a holistic approach, Morley says.

Attendees also wanted more targeted information about which specific home hazards are most dangerous to health. “There’s a lot one can do to improve a home environment—how can we help the workers prioritize? In the revised training, we will try to be more focused in terms of addressing critical hazards,” Morley says.

One of the attendees, David Brosch, chief inspector for the City of Baltimore Weatherization Program, says that the training reinforced some of the ideas his department is trying to apply. “We’ve gotten fairly good at insulating houses, but now we’re concerned about making them too air-tight, which can cause moisture or indoor air-quality problems,” he says. Brosch tries to address indoor air quality by, for example, testing furnaces and other appliances that can create combustion by-products. The pilot training also gave Brosch some insight into areas where further training would be useful, such as the causes of mold.

Brosch adds that workers will need practical tools to apply the theories of healthy housing. He is excited about a software tool that the training network is developing for use as a comprehensive checklist and reporting tool when visiting homes. Joe E. Beck, a professor of environmental health at Eastern Kentucky University, is leading development of the Hazard Assessment and Reduction Program for Housing, software that can be loaded onto an electronic inspection tablet or personal digital assistant. Once complete, the program will prompt workers to check for specific health and safety risks related to housing, and will be able to generate fact sheets on numerous topics using a portable printer. So when the inspection is done, the worker will be able to provide the residents with a summary report of findings as well as information on how to address identified deficiencies and hazards.

The challenge is translating this broader policy focus into practical action. “How can we get [public health and housing workers] to really change what they do back home?” Tohn says. “The training by itself will not change that. We have to start opening people’s eyes to [the holistic approach to healthy housing] and challenge them to go back and create systems that offer this one-touch approach.”

The training network is part of a call for change; it is one way to inspire workers in public health, environmental health, and housing to transform the system by changing what they do every day. “We hope the training center and network will contribute greatly to a change in mind-set,” Hershovitz says. “There’s a lot to be done. And this network is a darn good first step.”

## Figures and Tables

**Figure:**
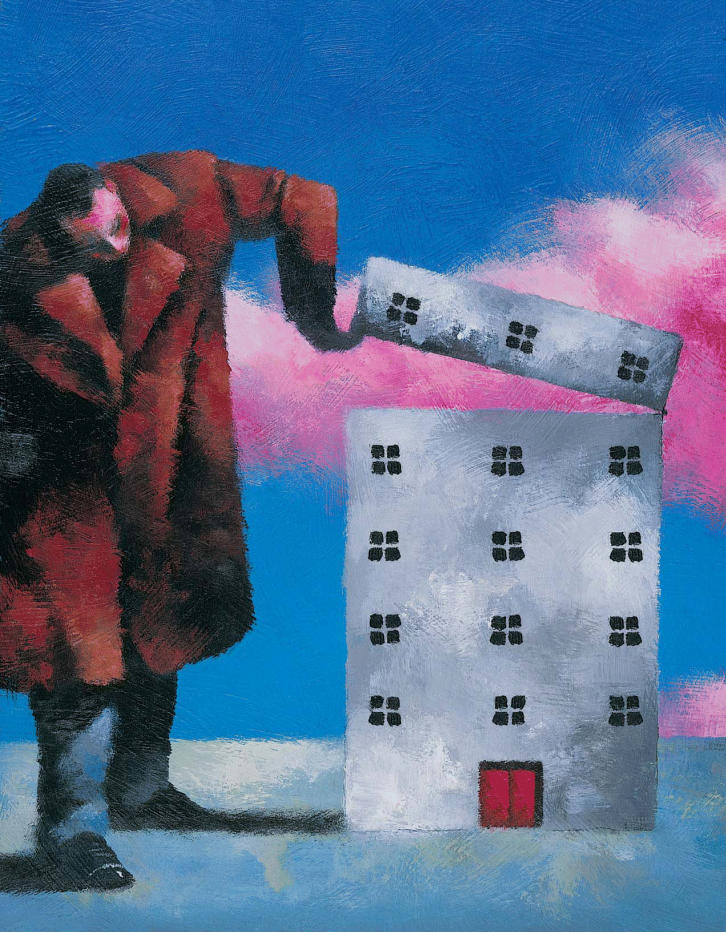


**Figure:**